# Diagnostic Accuracy of Ultrasonography for Knee Pathologies: A Prospective Comparative Study With MRI

**DOI:** 10.7759/cureus.104751

**Published:** 2026-03-06

**Authors:** Priyanka Priyanka, Rupali Sharma, Roopali Mangotra, Ruchika Bali

**Affiliations:** 1 Radiodiagnosis, Government Medical College and Hospital, Jammu, IND

**Keywords:** diagnostic accuracy, knee joint, magnetic resonance imaging, ultrasonography, ultrasound (usg)

## Abstract

Background

Knee joint pathologies are a common cause of orthopedic consultation, with many requiring imaging. Magnetic resonance imaging (MRI) is considered the non-invasive imaging reference standard with superior soft tissue resolution. However, it is expensive and time-consuming. High-resolution ultrasonography (HRUS), on the other hand, provides high soft tissue resolution for superficial structures, is cheaper, and is readily available with dynamic capabilities.

Methods

This prospective study was carried out on 50 patients referred from orthopedics to the radiology department of Government Medical College and Hospital, Jammu, with a request for MRI of the symptomatic knee joint. All patients underwent USG first, followed by MRI with standard knee protocol on a 1.5 T Siemens MRI (Munich, Germany). MRI was used as the reference standard. Ultrasonography (USG) findings were compared with MRI findings, and diagnostic indices, including sensitivity, specificity, accuracy, positive predictive value, and negative predictive value, were calculated.

Results

The anterior cruciate ligament and the medial meniscus were the most commonly damaged structures. HRUS showed low sensitivity (46.1% and 40%, respectively) but high specificity (95.8% and 97.5%, respectively) for medial and lateral meniscus injuries. Diagnostic performance of USG was excellent for superficial structures, including collateral ligaments, tendons, synovitis, collections, and popliteal cysts, with specificity approaching 100%. Joint effusion detection showed 94.7% sensitivity and 100% specificity. However, the cruciate ligaments were poorly visualized on USG.

Conclusion

Although MRI remains the gold standard for thorough evaluation of knee pathologies, USG has shown strong specificity and good diagnostic performance for superficial soft tissue structures. Therefore, USG may serve as a valuable screening tool, particularly in resource-limited settings.

## Introduction

The knee joint is the largest and one of the most important weight-bearing joints in the body. Mechanical stress in the knee increases while running, jumping, or carrying large objects. One of the most common mechanisms of knee damage is direct trauma, which is common in athletic injuries [1,2].

Arthroscopy is the definitive gold standard for diagnosing internal derangements of the knee. Magnetic resonance imaging (MRI) is considered the non-invasive imaging standard for diagnosing knee pathologies because of its higher soft tissue contrast, multiplanar capabilities, and lack of ionizing radiation. Several studies have suggested that MRI is equally reliable as arthroscopy in detecting ligament and meniscal lesions in the knee joint [3-5].

Nowadays, ultrasound (USG) is gaining recognition and becoming an integral imaging modality in the examination of the musculoskeletal system. The advantages of USG encompass its easy availability, cost benefits, dynamic evaluation of structures, and quick comparison with the opposite side. The primary strength of knee USG lies in the assessment of para-articular tissues [6]. It is preferable to use high-resolution USG (HRUS) as a preliminary investigation for various knee injuries, with MRI reserved for equivocal cases [7]. Moreover, many existing studies are retrospective, focus on isolated structures, or lack comprehensive diagnostic accuracy indices in a prospective comparison with MRI. Therefore, further prospective validation is warranted to better define the diagnostic scope and limitations of HRUS in routine clinical practice.

There is an ongoing need for standardization of USG techniques, validation against MRI, and expansion of dynamic point-of-care applications. This prospective study aims to evaluate the diagnostic performance of HRUS compared with MRI as the reference standard in symptomatic knee patients, by calculating sensitivity, specificity, predictive values, and overall diagnostic accuracy for individual knee structures.

## Materials and methods

Study design

An observational prospective study was conducted on patients referred from the orthopedic clinic to the Department of Radiodiagnosis, Government Medical College and Hospital, Jammu. All patients underwent USG examination of the knee joint, followed by MRI. The study was conducted over one year (November 2020 to October 2021) and included 50 patients. As this was a time-bound study, all consecutive patients fulfilling the inclusion and exclusion criteria during the study period were enrolled based on feasibility. Therefore, no formal prior sample size calculation was performed. Institutional ethical clearance was obtained before the commencement of the study (approval no. IEC/GMC/2020/211 dated October 28, 2020), and informed written consent was obtained from all participants. USG was performed by a senior consultant with 10 years of experience in musculoskeletal imaging. MRI examinations were interpreted independently by a separate radiologist with 5-6 years of experience in MRI reporting. At the time of USG examination, the radiologists were unaware of the specific MRI findings.

Inclusion and exclusion criteria

All symptomatic patients with knee problems such as pain, swelling, locking, limitation of movements, and external injuries were included. Patients with metallic implants, post-operative knee joints, and knee deformities and uncooperative patients were excluded.

USG technique

USG scanning was performed on a Mindray DC-70 Exp (Shenzhen, China) or SAMSUNG RS80 EVO (Seoul, South Korea) USG machine with a high-frequency (3-12 MHz) linear transducer. Anterior, medial, and lateral compartments of the knee were evaluated in the supine position with 20-30 degrees of flexion. In addition, external and internal hip rotation was done while evaluating the medial and lateral aspects of the knee, respectively. The posterior aspect of the knee was evaluated in the prone position with the knee extended [8].

MRI technique

Scans were acquired on a Siemens MAGNETOM Symphony 1.5 T MRI machine (Munich, Germany) using standard knee scanning protocol, including axial, coronal, and sagittal proton density fat suppression (PDFS) sequences, sagittal T1-weighted sequence, and sagittal T2-weighted sequence. Other sequences, such as short-tau inversion recovery (STIR), gradient-echo, three-dimensional PDFS, and post-contrast T1-weighted images, were used whenever required.

Statistical analysis

MRI was used as the reference standard, as arthroscopy was not ethically or clinically indicated in all patients. The statistical analyses of the results were performed using MedCalc statistical software (MedCalc Software Ltd., Ostend, Belgium). Diagnostic indices, including sensitivity, specificity, positive predictive value (PPV), negative predictive value (NPV), and overall accuracy, were calculated using 2 × 2 contingency tables.

## Results

Our study included a total of 50 patients, comprising 36 men (72%) and 14 women (28%). The majority among them were aged <20 years (28%), followed by 21-30 years (20%), 31-40 years (20%), 41-50 years (16%), and >50 years (16%) (Table 1).

**Table 1:** Age-wise distribution of cases.

Age (years)	Number of cases	Percentage
<20	14	28%
21-30	10	20%
31-40	10	20%
41-50	8	16%
>50	8	16%
Total	50	100%

The majority (66%) of patients had a history of trauma. Non-traumatic patients came with a history of swelling, pain, and fever.

On MRI, 47 patients had positive findings attributed to their complaints. The pathologies most frequently observed were involving the anterior cruciate ligament (ACL) (52%) and medial meniscus (MM) (52%), followed by joint effusion (38%) and lateral meniscus (LM) (20%) (Table 2). Among ACL injuries, partial ACL tears were most common. In 80% of cases, ACL tears were associated with posterior cruciate ligament (PCL) buckling. No PCL buckling was seen in the absence of ACL injury. In 69% of cases, ACL tear was associated with MM injury.

**Table 2:** Various knee structure pathologies detected on MRI (No. of cases = 50). MRI: magnetic resonance imaging; ACL: anterior cruciate ligament; PCL: posterior cruciate ligament; MCL: medial collateral ligament; LCL: lateral collateral ligament

Structures	MRI	Prevalence
ACL	26	52%
PCL	3	6%
MCL	5	10%
LCL	1	2%
Tendons (quadriceps & patellar)	2	4%
Medial meniscus	26	52%
Lateral meniscus	10	20%
Parameniscal cyst	6	12%
Joint effusion	19	38%
Synovial proliferation	6	12%
Popliteal cyst	4	8%
Collection	2	4%
Fracture/contusion/erosion	9	18%
Chondromalacia patella	1	2%
Osteomyelitis	1	2%
Soft tissue tumor	1	2%
Bone tumor	1	2%

On USG, the most commonly detected abnormality was joint effusion in 18 cases. For MM and LM injuries, USG showed low sensitivity (46.1% and 40%, respectively) but high specificity (95.8% and 97.5%, respectively). The USG demonstrated high specificity for superficial structures, including collateral ligaments, tendons, synovitis, collections, and popliteal cysts, with specificity approaching 100%. Joint effusion detection showed 94.7% sensitivity and 100% specificity. One false-positive LM and MM tear was seen on USG (Table 3). Due to limited visualization of cruciate ligaments on USG, statistical evaluation of diagnostic indices for ACL and PCL was not feasible.

**Table 3:** Diagnostic accuracy of USG compared with MRI. Values in parentheses represent 95% confidence intervals (CIs) calculated using exact binomial methods. PPV: positive predictive value; NPV: negative predictive value; USG: ultrasonography; MRI: magnetic resonance imaging; MCL: medial collateral ligament; LCL: lateral collateral ligament

Structures	True +ve	False +ve	False -ve	True -ve	Sensitivity (%) (95% CI)	Specificity (%) (95% CI)	PPV (%) (95% CI)	NPV (%) (95% CI)	Accuracy (%)
Medial meniscus	12	1	14	23	46.1 (26.5-66.6)	95.8 (73.0-98.9)	92.3 (59.9-96.0)	62.1 (51.9-69.5)	70
Lateral meniscus	4	1	6	39	40 (12.1-73.7)	97.5 (86.8-99.9)	80 (33.3-96.9)	86.6 (79.6-91.5)	86
Parameniscal cyst	5	0	1	44	83.3 (35.8-99.5)	100 (91.9-100)	100 (47.8-100)	97.7 (88.0-99.6)	98
MCL	5	0	0	45	100 (47.8-100)	100 (92.1-100)	100 (47.8-100)	100 (92.1-100)	100
LCL	1	0	0	49	100 (2.5-100)	100 (92.7-100)	100 (2.5-100)	100 (92.7-100)	100
Tendons	2	0	0	48	100 (15.8-100)	100 (92.6-100)	100 (15.8-100)	100 (92.6-100)	100
Popliteal cyst	4	0	0	46	100 (39.7-100)	100 (92.2-100)	100 (39.7-100)	100 (92.2-100)	100
Joint effusion	18	0	1	31	94.7 (73.9-99.8)	100 (88.7-100)	100 (81.4-100)	96.8 (82.1-99.5)	98
Synovitis	6	0	0	44	100 (54.0-100)	100 (91.9-100)	100 (54.0-100)	100 (91.9-100)	100
Collection	2	0	0	48	100 (15.8-100)	100 (92.6-100)	100 (15.8-100)	100 (92.6-100)	100

**Figure 1:**
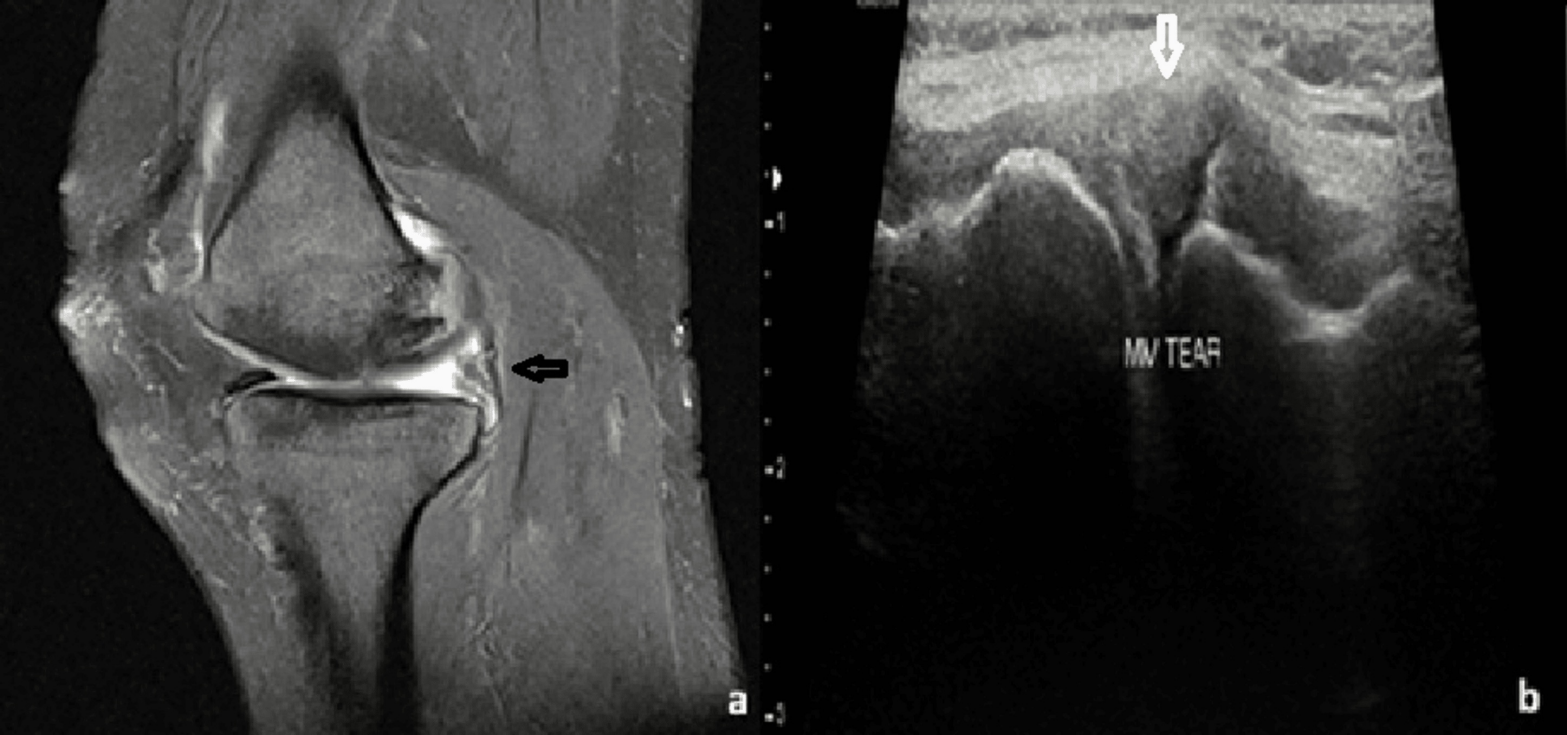
A 24-year-old male patient with a radial tear of the medial meniscus. (a) Sagittal PDFS MRI image demonstrating a radial tear involving the posterior horn of the medial meniscus, showing the characteristic “ghost sign.” (b) Corresponding USG image demonstrating a hypoechoic wedge-shaped area within the normal echogenic meniscus, consistent with a tear. PDFS: proton density fat suppression; USG: ultrasonography; MRI: magnetic resonance imaging

**Figure 2:**
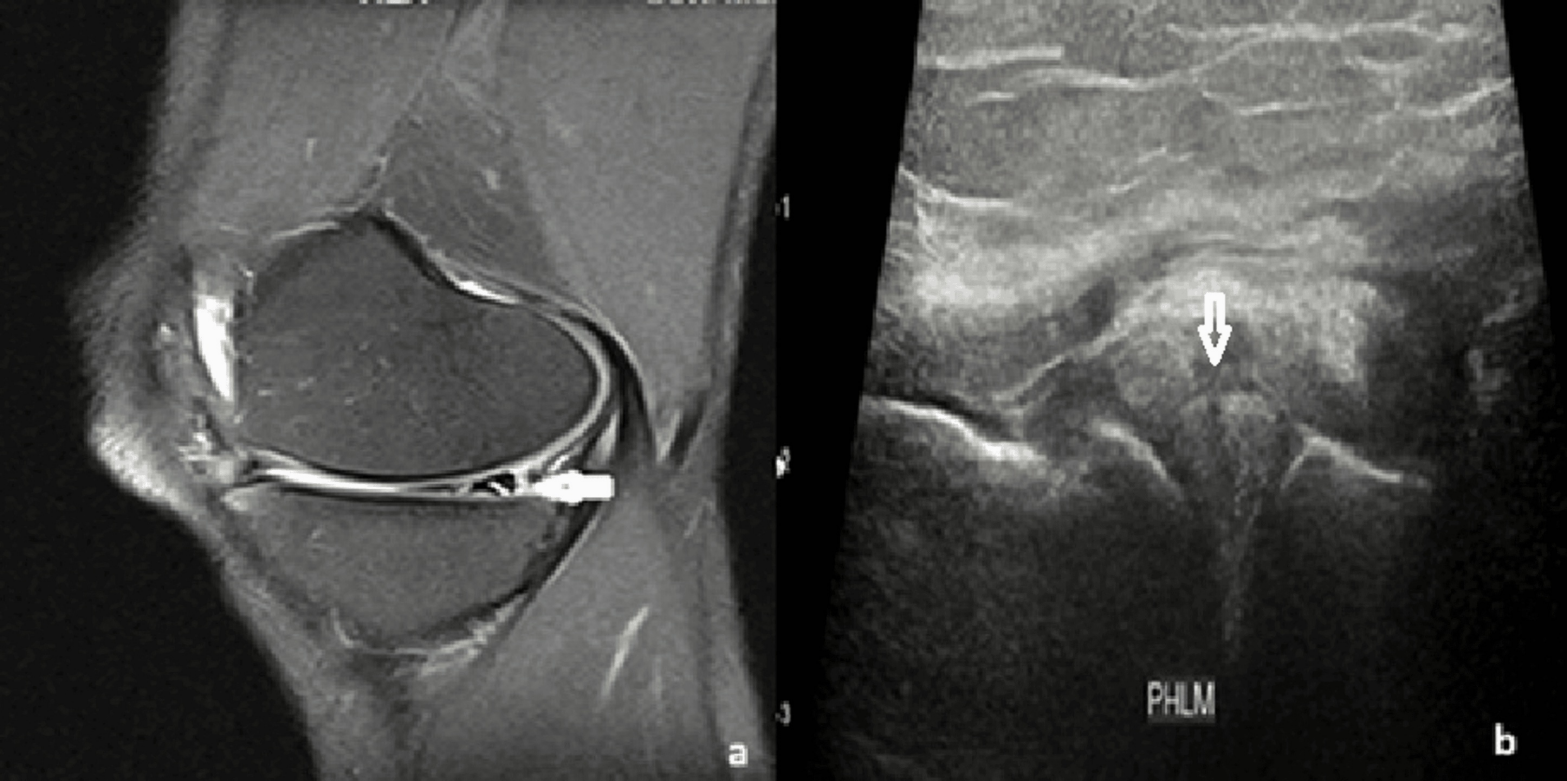
A 21-year-old male patient with a vertical tear of the lateral meniscus. (a) Sagittal PDFS MRI image demonstrating a vertical tear extending to the inferior articular surface of the lateral meniscus. (b) Corresponding USG image demonstrating a linear hypoechogenicity within the normally echogenic lateral meniscus, consistent with a tear. PDFS: proton density fat suppression; USG: ultrasonography; MRI: magnetic resonance imaging

**Figure 3:**
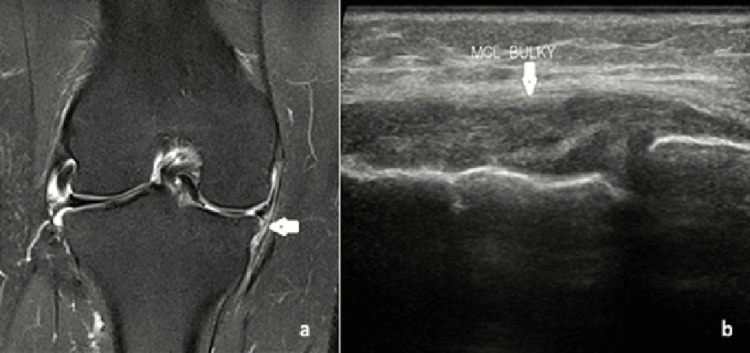
A 30-year-old female patient with a partial tear of the medial collateral ligament (MCL). (a) Coronal PDFS MRI image demonstrating thickening and hyperintensity in the deep fibers of the MCL, suggestive of a partial tear. (b) Corresponding USG image showing a bulky and hypoechoic MCL. PDFS: proton density fat suppression; USG: ultrasonography; MRI: magnetic resonance imaging

**Figure 4:**
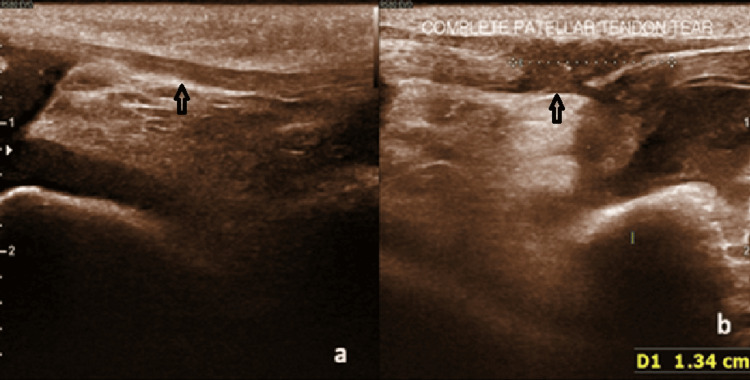
Ultrasonographic (USG) appearance of normal and torn patellar tendon. (a) USG image of a 32-year-old male showing a normal patellar tendon with preserved fibrillar echotexture and continuity. (b) USG image of a 28-year-old male demonstrating a complete patellar tendon tear with breach in tendon continuity.

Meniscal tears on USG appeared as linear or wedge-shaped hypoechoic areas within the normal echogenic meniscus. Representative cases, including one radial tear with a characteristic “ghost sign” on MRI and another vertical tear with its corresponding USG images, are shown in Figures 1, 2. Collateral ligament injuries included one complete and four partial tears. A partial tear of the medial collateral ligament (MCL) appeared as ligament thickening and hyperintensity on MRI and as a bulky, hypoechoic ligament on USG (Figure 3). One case of complete patellar tendon tear was seen in our study, with a representative USG appearance of a normal and torn patellar tendon illustrated in Figure 4. A normal tendon demonstrated preserved fibrillar echotexture, whereas a complete tear shows disruption of tendon continuity (Figure 4).

## Discussion

Although arthroscopy is the definitive gold standard for internal derangements of the knee, MRI was used as a reference to compare the diagnostic accuracy of USG in this study due to its non-invasive nature and widespread clinical use. Also, MRI is the most appropriate screening tool before a therapeutic procedure [9]. Based on the high predictive value of a negative MRI, it is useful to exclude patients from unnecessary arthroscopy [10].

Meniscus

MM injury was more frequently observed than LM, which is consistent with Singh et al., who also showed that MM injury (52%) is more common than LM injury (22%) [11]. For MM tears, USG showed high specificity (95.8%) and PPV (92.3%), suggesting it is reliable for confirming tears; however, it showed low sensitivity (46.1%); therefore, it is not reliable for ruling out pathology. For LM tears, USG demonstrated low sensitivity (40%) and high specificity (97.5%), indicating that a positive finding is reliable, but a negative finding does not confidently exclude pathology. Although the sensitivity of USG for meniscal tears was moderate in our study, the specificity was high. Elshimy et al. also demonstrated variable sensitivity but good overall diagnostic agreement with MRI for meniscal injuries, suggesting that USG may be particularly useful as a screening tool rather than a definitive diagnostic modality [12]. The low sensitivity may be explained by difficulty in detecting deep, root, and bucket handle tears [13,14]. The cystic lesions encountered in association with meniscal tears were meniscal or parameniscal cysts and were seen in five cases. In one case, the parameniscal cyst was seen without an underlying meniscal tear. This is consistent with Wu et al., who found a link between the development of meniscal tears and parameniscal cyst formation [15].

Cruciate ligaments

ACL was found to be one of the most commonly injured knee structures, with partial tears being more common than complete tears in our cohort. Frobell et al. demonstrated that ACL injury is the most common in acute knee trauma, consistent with our result [16]. However, in contrast, a complete tear was most common in their study. PCL buckling was observed in 80% of ACL tears. Polat et al. concluded that PCL buckling is not specific to ACL tear [17]. However, in our study, no PCL buckling was found in the absence of ACL injury. PCL tears were uncommon in our cohort (6%), consistent with Sonin et al., who found only 2.6% cases of PCL injuries in their study [18]. The cruciate ligaments were poorly visualized on USG due to their deep intra-articular location and lack of proper penetration of the USG beam.

Tendons, collateral ligaments, and surrounding soft tissue

The USG demonstrated excellent performance in detecting tendon tears. USG detected joint effusion with 100% specificity and PPV. Diagnostic performance of USG was excellent for superficial structures, including collateral ligaments, tendons, synovitis, collections, and popliteal cysts, with specificity approaching 100%. This is comparable to Maheshwari et al., who demonstrated perfect agreement between USG and MRI for detecting Baker’s cyst and near-perfect agreement for detecting joint effusion, soft tissue edema, and osteophytes [19]. The detection of knee effusion by sonography may indicate internal knee derangement in trauma patients [20]. USG also showed excellent diagnostic performance in detecting synovitis/synovial proliferation. It is also useful to guide the aspiration of joint fluid and synovium.

Osseous

In our study, osseous injuries were found in 16 patients. Most of these were bony contusions involving the tibial and femoral condyles. The pattern of bone contusion after trauma can help determine the type of injury that occurred [21]. On USG, one of our cases demonstrated a tiny chip avulsion fracture measuring 3.5 mm along the femoral attachment of the MCL. This small chip fracture was not identified on MRI because of its tiny size, less than the slice thickness. USG may detect small superficial cortical abnormalities that can be less conspicuous on MRI.

Although specificity was high for several structures, the wide confidence intervals reflect the limited sample size and should be interpreted cautiously. Rather than concentrating just on isolated traumatic injuries, our study encompassed a heterogeneous symptomatic group, and yet, this approach mirrors actual clinical referral patterns. Consequently, this provides valuable insights about the performance of USG in a variety of common knee conditions.

Limitations

The study’s statistical power may have been constrained by the small sample size, especially for less frequent pathologies. This increases the potential risk of Type II error. The small sample size may have contributed to the high specificity observed for some structures. Large multicentric studies would be necessary to validate these findings. MRI was used as the reference standard, as arthroscopy was not available in all cases. Interobserver variability was not formally assessed, which may affect reproducibility. Despite experienced operators, USG remains inherently operator-dependent, which may affect generalizability.

## Conclusions

USG is a cost-effective and readily available imaging modality for evaluating knee joint structures with promising results. USG demonstrated high accuracy and PPV in evaluating the superficial soft tissue structures, such as tendons, collateral ligaments, superficial sections of the meniscus, joint effusion, synovial proliferation, and popliteal and parameniscal cysts. However, statistical evaluation was not possible for deep intra-articular structures, particularly the cruciate ligaments and deep parts of the menisci, because of inadequate visibility.

While MRI remains the recommended imaging modality for thorough evaluation of internal derangements of the knee, USG may serve as a valuable screening tool, especially in resource-limited settings. A screening modality with high specificity may allow prioritization of patients requiring advanced imaging, thereby optimizing resource utilization. To further evaluate these results and standardize the USG assessment methods, large multicentric research is necessary.
